# Health care Professionals' experiences of counselling competence in digital care pathways – A descriptive qualitative study

**DOI:** 10.1002/nop2.1729

**Published:** 2023-03-24

**Authors:** Juulia Kaihlaniemi, Pia Liljamo, Mira Rajala, Pirjo Kaakinen, Anne Oikarinen

**Affiliations:** ^1^ Unit of Health Science and Technology University of Oulu Oulu Finland; ^2^ Administrative Centre Oulu University Hospital Oulu Finland

**Keywords:** competence, counselling, digital care pathways, eHealth, health care professional, qualitative research

## Abstract

**Aim:**

Health care professionals are tasked with the role of supporting patients in using eHealth services in their personal care and counselling competence in digital environments to ensure appropriate patient care. Our aim was to describe health care professionals' experiences of counselling competence in Digital Care Pathways.

**Design:**

A descriptive qualitative study.

**Methods:**

Interviews with semi‐structured questions were conducted. Twelve healthcare professionals were interviewed. The data were analysed using inductive content analysis. The results were reported according to the Consolidated Criteria for Reporting Qualitative Research.

**Results:**

The analysis revealed eight distinct areas of competence related to counselling in Digital Care Pathways, namely, counselling competences related to the use of Digital Care Pathways, supporting patients' self‐care, information technology competence, competence in creating an interactive counselling relationship on the Digital Care Pathway, information management, ethical competence related to counselling in Digital Care Pathways, competence to developing Digital Care Pathways services, and change competence.

**No Public or Patient Contribution:**

Due to the complex and unpredictable circumstances of COVID, people's strict confinement in the hospital prohibited free access to them and the study environment. Therefore, the professionals involved in the study were interviewed through online systems.

## 1 INTRODUCTION

The digitalization of health care services is rapidly increasing on a global level. This rapid digitalization has created new opportunities for communication between health care professionals (HCPs) and patients (Koivunen & Saranto, 2018; Odendaal et al., 2020). For instance, eHealth services can now be implemented remotely and are defined as the utilization of digital technologies to promote health. eHealth is an umbrella term for a range of different technologies, example, remote reception solutions, mobile applications, or various patient portals (Koivunen & Saranto, 2018; Öberg et al., 2018). Thus, HCPs are now able to readily reach patients who live in geographically distant locations and employ effective patient‐professional communication to improve care quality and the coordination of services (Koivunen & Saranto, 2018; Odendaal et al., 2020). Furthermore, many patients are now skilled at using eHealth services, and actively search for health‐related information and monitor their health records online (Koivunen & Saranto, 2018; Öberg et al., 2018).

The rapid digitalization of health care has transformed HCPs work content and responsibilities (Odendaal et al., 2020). The role of an HCP is to support patients in utilizing eHealth services for their personal care (Jarva et al., 2022; Kinnunen et al., 2019). An HCP's digital health competence is related to their ability to provide patient‐centered care through different digital channels, use digital health systems and technology, and interact with the patient through digital means (Jarva et al., 2022). A systematic review performed by Paalimäki‐Paakki et al. (2022) concluded that digitalized counselling environments are comparable with standard counselling methods; this suggests that the use of digital counselling environments could accompany standard counselling methods.

The advent of digitalization in health care has made technology‐related competence critical to nurses' work (van Houwelingen et al., 2016). In other words, the digital health competence of HCPs ensures patient safety (Jarva et al., 2022; Öberg et al., 2018). In clinical nursing, the five most important competence areas are nursing documentation, data protection and security, information and knowledge management in patient care, ethics and information technology, and quality assurance and management (Egbert et al., 2019). In addition to technological skills, a professional's attitudes and beliefs regarding the use technology has is a considerable influence on their competence in digitalization (Konttila et al., 2019). Furthermore, a HCP's competence in eHealth involves the evaluation of eHealth services and combine traditional and digital methods (Jarva et al., 2022). In other words, eHealth services are not necessarily suitable for all patients, and some would also benefit from traditional face‐to‐face counselling (Koivunen & Saranto, 2018; Odendaal et al., 2020). For example, according to Akinosun et al., (2021), the use of digital health interventions did not lead to improvements in all lifestyle risk factors among cardiovascular patients relative to usual care interventions. Therefore, HCPs should be able to determine patients' skills, attitudes and readiness to use digital solutions when considering counselling via eHealth systems (Koivunen & Saranto, 2018). HCPs need new competences when transitioning to digital environments and counselling patients via eHealth services (Kinnunen et al., 2019; Konttila et al., 2019).

Communication and counselling skills are generally interrelated competences in health care, as well as being statistically significant to health promotion and disease treatment in eHealth services (Konttila et al., 2019). A professional can ensure that a patient understands their situation and the treatment process by asking specific questions and tailoring the counselling on an individual level. Problem‐solving skills are also important when clinical experience and technology‐related decision‐making are combined (van Houwelingen et al., 2016). It has been recognized that a variety of digital counselling environments have potential in motivating self‐care, providing peer support through interaction, and offering reliable, understandable, and up‐to‐date information to help patients with both their disease management and adherence to self‐care (Frederix et al., 2015).

HCPs who show a positive attitude and patience will encourage patients to take an active role in their personal care (van Houwelingen et al., 2016). According to previous studies, HCPs often report negative experiences about using technology in their work; for this reason, certain HCPs have rather negative attitudes to digitalization (Koivunen & Saranto, 2018; Konttila et al., 2019). Therefore, HCPs may feel that they possess insufficient technological skills even if they fluently use digital services in their personal lives (Koivunen & Saranto, 2018). HCPs have also reported insufficient skills in developing digital health services (Öberg et al., 2018). A professional can improve their digital competence by integrating learning into daily work and highlighting the benefits of technology (Konttila et al., 2019).

The five Finnish university hospitals have developed Health Village patient portal, which includes Digital Care Pathways (DCP), to supplement traditional care paths for different patient groups. DCPs include information about a specific disease, support in self‐care, frequently asked questions, and various relevant assignments and queries. Furthermore, patients who use the service will have the opportunity to self‐monitor their progress and assess symptoms. The patient can also use the service to remotely meet with their HCP, either through secure messaging or video conferencing. (Kujala et al., 2020; Liljamo et al., 2020, 2021).

According to a Finnish national health record survey study, nurses demonstrate a satisfactory level of general IT competences, including basic IT competence, data protection and security, and ethical knowledge. On the other hand, expertise related to patient work in a digital environment is at a weaker level (Kinnunen et al., 2019). Consequently, it is necessary to clarify HCPs' views on the topic. The aim of this study was to describe HCPs' experiences of counselling competence in DCPs. The study answered the following question: Which competences do HCPs need when they are counselling patients in DCPs?

## 2 METHODS

### 2.1 Study design

A descriptive qualitative interview study was chosen to provide deeper knowledge concerning HCPs' perspectives of their counselling competence in DCPs. The study results were reported using the Consolidated Criteria for Reporting Qualitative Research (COREQ) checklist (Tong et al., 2007; Supplementary File S1).

Various occupational groups of HCPs, such as nurses, midwives, rehabilitation instructors, physiotherapists, and occupational therapists, work in DCPs. In this study, a HCP refers to any professional representing these occupational groups. HCPs from one university hospital were recruited using convenience and snowball sampling (Polit & Beck, 2017). To be eligible to participate, the HCP had to have experience of counselling patients during at least the pilot phase of DCP. The participants were recruited with the assistance of the development manager of Health Village, who had access to the administrators of the DCPs, and their supervisors. In addition, the researcher advertised the study during a remote meeting with the administrators of DCPs. Potential participants were approached by an email which included information about the study and the researcher's contact information if an HCP was interested in participating. Following the sending of an email that stated interest to participate, each HCP signed a consent form before the interview was conducted. Participants had the opportunity to ask additional questions at the beginning of the interview.

### 2.2 Data collection

The first author (blinded for the review) interviewed 12 HCPs from February – March 2021. The interviews were conducted through video conferencing because of the COVID‐19 pandemic. Participants were interviewed at their workplace. Data collection was completed when saturation was achieved, that is, no new information of new dimensions or theoretical categories were obtained from the interviews with additional participants, and the data were classifiable (Kyngäs et al., 2020).

Data saturation was followed by an analysis that was started during the interviews. The semi‐structured interviews were completed with each participant. The interview guide included the following nine topics: 1. Counselling competences in digital environment; 2. Patient orientation in digital counselling; 3. Communication skills; 4. Technological skills; 5. Competence in information management; 6. Ethical knowledge; 7. Competence in developing a DCP; 8. Organizational and collegial support; and 9. Attitudes towards digital counselling. Participants responded to the seventh topic if they had experience in developing DCP. (Polit & Beck, 2017.)

The selected topics were based on previous research and the first researcher's understanding about the subject. The content of the interview guide was evaluated by two HCPs before the interviews. Similar sub‐questions were deleted based on the findings of the evaluation. Furthermore, one pilot interview was conducted and included in the study data. The interviews were video recorded with the permission of the participants, after which they were transcribed into text format and checked for accuracy. The transcription process generated 94 pages of text (Times New Roman, font size 12, line spacing 1). The interviews lasted an average of 47 min and ranged from 33–84 min. Field notes were made after each interview.

### 2.3 Data analysis

The data were analysed following the guidelines of inductive content analysis (Kyngäs et al., 2020). Prior to the actual analysis, the written material was read through several times so that the researcher became familiarized with the data. Next, a unit of analysis was chosen; in this case, the unit of analysis was sentences that corresponded to the research question. These sentences were then classified as open codes. The open codes were analysed and grouped together to form sub‐concepts. In the next step of data abstraction, the sub‐concepts were categorized into concepts based on similarities in content. In the final step, main concepts were formed from the identified concepts (Kyngäs et al., 2020). The main concepts which described HCPs' experiences of counselling competences in DCPs were then reported (Elo & Kyngäs, 2008; Kyngäs et al., 2020). The formation of a sub‐concept as an example of content analysis is described in Table 1.

**TABLE 1:** Example of the content analysis process.

Unit of analysis	Open code	Sub‐concept
One can involve patients in such a way that they follow their daily lives in relation to challenges. (A2)	Patients' involvement	Expertise in supporting patients' self‐care
They are chronically sick children, so the motivation of the family and the motivation of the child are very important for self‐care. (A7)	Motivation is important for self‐care	
We're not shepherding, we are going to get the patient involved in his/her own care. (A8)	Patient takes responsibility for his/her self‐care	
Know how to guide self‐care methods. (A8)	Self‐care counselling	
Patients' self‐care and lifestyle counselling. Vaccinations, oral hygiene, exercise, smoking, alcohol consumption. (A9)	Information about self‐care	
Self‐care is probably the most important thing what needs to be known. (A10)	Self‐care knowledge	
Themes related to counselling are mainly peristaltic action, urination, skin care, nutrition, sleep, and sexuality. (A12)	Themes related to counselling	

### 2.4 Ethics

The presented research followed the ethical guidelines of the Helsinki declaration (World Medical Association, 2013). The study protocol was reviewed and approved according to practices of the local hospital district; furthermore, the head nurses gave their permission for the research to be conducted. According to legislation in Finland, Research Ethics Committee approval was not required since the study does not involve minors, direct or indirect physical or physiological harm to the participants, or clinical trials. (Medical Research Act No. 488/1999).

The participants received information about the study and its aims before being asked for written informed consent. The participants were also informed that the interview would be video recorded, and that only their speech would be analysed. Participation was voluntary and the participants could withdraw from the study at any time without consequences. The study also complied with the European Union's General Data Protection Regulation (GDPR, 2016). Only researchers had access to the collected data, which are stored on the first researcher's (blinded for the review) computer in a password‐protected folder until the data are published. All of the participants' personal information was kept confidential, and their anonymity was ensured when reporting the findings. Participants were treated fairly and equally during the research (Pietilä et al., 2020).

## 3 RESULTS

### 3.1 Study participants

All of the participants (*n* = 12) were women who were between 36–61 (mean 47) years of age. The participants had work experience ranging from 10 to over 25 (mean 19.4) years; they had a maximum of 2 years of experience in working on the DCP. The participants' demographics are presented in detail in Table 2.

**TABLE 2:** Demographic information of the study participants (*n* = 12)

Characteristic	Frequency (%)
Age (Mean 47 years)	
36–50 years	8 (67%)
50–61 years	4 (33%)
Gender	
Female	12 (100%)
Male	0 (0%)
Professional title	
Registered nurse	11 (92%)
Other professional title	1 (8%)
Work experience (19,4 years)	
10–14 years	2 (17%)
15–19 years	4 (33%)
20–24 years	5 (42%)
>25 years	1 (8%)
Work experience in Digital Care Pathway	
<1 year	2 (17%)
1–2 years	7 (58%)
>2 years	3 (25%)
Context of the Digital Care Pathway	
Short‐term treatment	2 (17%)
Long‐term care	10 (83%)
Pathway for therapy	0 (0%)
Experience in developing Digital Care Pathway	
Yes	7 (58%)
No	5 (42%)

### 3.2 Health care professionals' experiences of counselling competence in digital care pathways

A total of eight main concepts and 19 sub‐concepts describing HCPs' experiences of counselling competence in DCPs were identified through inductive content analysis (Table 3). The results are described in the following sections.

**TABLE 3:** Health care professionals' experiences of counselling competence in Digital Care Pathways (boarders left for the review)

Main concept	Concepts	Sub‐concepts
Counselling competence related to the use of DCPs	Expertise in patient counselling regarding the use of DCPs Problem‐solving skills related to the use of DCPs	Skills for counselling related to the use of DCPs Knowledge about guidance material related to the use of DCPs Solving challenges related to the use of DCPs Skills in assessing patient's motivation, competence and tools
Counselling competence to support patients’ self‐care	Expertise related to the treatment of disease and the patient group Skills for involving patients in their self‐care	Knowledge on the subject Information about the treatment of the disease and the patient group Knowledge related to medical treatment Expertise in supporting patient's self‐care Counselling skills related to health care services
Information technology competence	Basic technology skills Expertise in using the DCP application	Basic information technology skills DCP application use is considered one of the basic skills Skills in using the DCP application Knowledge relevant to solving technical problems
Competence in creating an interactive counselling relationship via DCPs	Skills to motivate patients Communication skills in the digital environment Professional skills in the evaluation of messages	Expertise in patient‐centered counselling Motivational skills similar to nursing Skills to meet the patient on DCPs Encouraging communication Clear communication Empathetic communication Professional role in counselling via DCPs Ensuring the patient's understanding Time management skills
Information management competence related to counselling via DCPs	Skills in documenting counselling in the patient health record Skills in utilizing patient information Data searching skills in counselling	Documenting contact‐related matters Documenting changes in the treatment of the disease Transferring patient‐submitted data Documenting the information of counselling Utilizing the data in the patient health record Expertise to assess patient data on DCPs Using evidence‐based information Multidisciplinary competence Retrieving reliable information from other data sources
Ethical competence related to counselling via DCPs	Data privacy and security knowledge Ethical knowledge related to counselling	Reliance in the security of DCPs Awareness of the importance of security and security issues Communication skills to ensure data protection Careful work when processing personal data Professional skills in transferring data to the patient health record Awareness of the voluntary nature of the service Respect for the patient Using evidence‐based information Common practices in challenging counselling situations
The implementation of DCPs requires competence in developing services	Nursing expertise in content production Technological skills in content production Skills to support the involvement of DCP users	Extensive content production expertise related to different patient groups Identifying patient group challenges in content production Multidisciplinary expertise in content production Producing content requires a special type of technological know‐how Considering accessibility in content production Skills for patient involvement Skills in the orientation of professionals
The implementation of DCPs requires change competence	Positive attitude towards change Awareness of the significance of counselling in DCPs	Positive attitude towards learning new things Positive attitude towards change Counselling in DCPs was useful Communication between patient and professional became easier

#### 3.2.1 Counselling competence related to the use of digital care pathways

HCPs require counselling competence related to the use of DCPs. This involves expertise in patient counselling, along with problem‐solving skills relevant to the use of DCPs.

Counselling related to the use of DCPs was either conducted in the clinical setting at the hospital or on the DCP secure message application. The participants also felt that HCPs require knowledge about which guidance materials are related to the use of DCPs.

Knowledge about the usability of the digital care pathway, so I can guide the family. For example, being able to present it as: ‘Hi! This question is related to the frequently asked questions of the digital care pathway. So, you can check it now or come back to it later.’ (A3).

All of the instructions and how we bring the families into the digital care pathway, so that it would be tempting and easy. It makes it easier to do business if they don't think it is difficult. (A5).

Problem‐solving skills were needed if the patient had never used the service or had challenges using the application. As such, HCPs needed to be able to assess a patient's motivation, competence, and tools. For example, a patient's age, cognitive level, functional capacity, and technological competence had to be considered.

You can conclude from those messages whether the issue has been understood. So, you can guide and highlight, also in the digital care pathway, where to go and what kind of things should be put there. (A7).

#### 3.2.2 Counselling competence to support patients' self‐care

HCPs must also have the counselling competence necessary to adequately support patients' self‐care. This requires expertise related to the treatment of disease and various patient groups, along with skills in involving patients in their self‐care.

To sufficiently support self‐care, HCPs need information about the illness and its treatment, and expertise in identifying challenges related to self‐care. The participants highlighted knowledge related to medical treatment, such as information on the effects of a medicine, correct dosage, possible side effects, and blood tests.

My own knowledge, what I have gained over the years. So, I have been able to use it. I can answer then. (A7).

You must know something about medical treatment. If the patient uses lots of opiates, then you can consider the need for laxatives. (A12).

Involving the patient in their own care requires expertise in supporting the patient's self‐care and counselling skills related to health care services. The most common topics in self‐care counselling were vaccinations, physical activity, smoking and alcohol consumption, urination and peristaltic action, nutrition, skin care, oral health, sleeping and sexuality. Patients were informed about the available health care services and also encouraged to seek peer support.

One can involve patients in such a way that they follow their daily lives in relation to possible challenges. (A2).

How the patient can receive, for example, more information and peer support, along with the possibility for some forms of rehabilitation. (A9).

#### 3.2.3 Information technology competence

Information technology competence is a requirement for counselling in DCPs. More specifically, HCPs who participate in digital environments need both basic technology skills and expertise in using DCP applications.

Basic information technology skills, such as managing access to computers and different applications, facilitated counselling in DCPs. The ability to use DCP applications was considered one of the basic skills.

It is easier when it is relatively natural to use different programs. When you in a way know, how they are talking to each other. (A8).

Counselling in DCPs requires the HCP to be able to log in to the DCP application, connect with patients, and use the application functions. For this reason, practices related to technical problems in the DCP application were also important.

You must know how to find the Digital Care Path; this is because we register blood tests on the calendar. You also have to know how to check the messages. (A11).

In those problem situations, (you know) where to connect. (A6).

#### 3.2.4 Competence in creating an interactive counselling relationship via digital care pathways

This main concept included several prerequisites, namely, skills to motivate patients and communication skills in digital environments. In addition, it was reported that HCPs must be able to evaluate the messages they receive.

Expertise in patient‐centered counselling is key for the HCPs who work in digital environments. The participants noted that identifying patient challenges and counselling demands serve as the basis for individualized counselling. Patients were encouraged to contact their HCP via the DCP if something about their treatment was unclear. The motivation skills needed for counselling in DCPs were similar to those present in traditional nursing.

It's not like thinking so much as a professional. You are considering the patient's position, what kind of counseling and advice you want to get so you can move forward. (A1).

Quite the same motivational skills as in this job anyway. (A7).

HCPs needed skills to meet the patient via the DCPs. Communication on DCPs was considered to be informal, personal, friendly, and polite. However, this form of communication required reflection because it was impossible to read the patient's facial expressions and gestures. Communication in the DCPs had to be encouraging, clear and empathetic. The latter was considered challenging even though the participants felt that it was important to emotionally support the patient. The participants shared that it was useful to ask additional questions related to the patient's coping in order to meet the related challenges and patient's concerns.

You must consider how to write those things, because it is often easier to explain face‐to‐face (A5).

You need to learn how to produce writing that is easy to read and encouraging, yet also brief. (A8).

Quite often, young people are afraid of the side effects of medicines. So, in a way, supporting them in those things. Some of them are very suspicious. (A11).

HCPs participated in the DCPs according to their professional role. Professionalism was needed to ensure patient understanding. Hence, the HCPs asked patients specific questions via the DCPs to ensure that they understood their care path. Assessments of the need for counselling required professional skills, especially in situations where it was difficult to tell whether the patient needed counselling by phone or at the health centre. Time management skills were needed because the HCPs had limited time to reply to messages.

You are here as a nurse and your job is to answer questions professionally. (A6).

I send a message which includes is a clarifying question. Based on the response I will know whether the patient requires more extensive counseling. (A8).

Time prioritization skills. In my opinion, that is one of the things you should think about. This includes when to respond, for example, can you respond now in only five minutes, or should you save this task for later when you can focus on counseling a little bit better. (A1).

#### 3.2.5 Information management competence related to counselling in digital care pathways

HCPs need to be able to document counselling in the patient health record and utilize patient information in counselling. The participants also felt that certain data searching skills were relevant to counselling.

HCPs need to document contact‐related matters and changes in the treatment of a disease in the patient's health record. Patient‐submitted data must also be transferred to the patient health system and documented in the patient report. Information regarding counselling provided via DCPs was documented in the patient health record using the following structure: the needs; goals; implementation; and results. The participants acknowledged that documentation in the patient information system was more formal than communication via DCPs.

If I write on the patient health record, that is where I should emphasize the essential health information issues related to the contact (A3).

You must document any cases in which the patient has consulted the doctor to change the medication. (A1).

You need to know the difference. What are the official nursing documents and the environment for the documentation and then what difference it makes for the DCP. (A8).

The participants felt that utilizing data in the patient health record helped HCPs answer the patients' individual questions. Expertise was also needed to assess patient data available in DCPs. Professional skills were significant in the assessment of symptoms or problems reported by the patient.

HCPs must be able to retrieve information from the information system. (A10).

The messaging that comes from patients can be descriptive. We may also prepare ourselves to respond to certain kinds of issues at the appointment. Then the patient would also be heard better. (A2).

I can react to whatever needs to be handled. For instance, what I must do or clarify if the child has diseases or some health‐related issues. (A5).

Evidence‐based information was utilized in counselling via DCPs. The participating HCPs mentioned that they sought information from the open pages of the Health Village portal, different databases, and treatment recommendations. Information related to the illness was found on DCPs. Moreover, HCPs could seek information from other sources that were found to be reliable. When discussing how to handle challenging issues, the HCPs emphasized the importance of multidisciplinary competence.

As a professional, you need to know where to get that reliable information. (A1).

Consulting a doctor or someone wiser, an expert. Usually another professional will ask you what you have been doing, and what should now be done with this patient's case? And there it comes again, the individuality. (A6).

#### 3.2.6 Ethical competence related to counselling via digital care pathways

In the context of DCPs, ethical competence comprised data protection, security knowledge, and ethical knowledge related to counselling.

HCPs relied on the data security of DCPs. However, awareness of data privacy and security challenges were considered important. The participants mentioned that in some cases it was difficult to ascertain where the information was coming from; hence, when a matter was sensitive and/or intimate, they may personally contact the patient by phone. Thus, communication skills were essential to ensuring data protection. HCPs were aware of the risks associated with the processing of personal data, and reported that each professional had to be particularly careful when processing these type of data. Another aspect of ensuring patient safety was the ability to transfer data obtained via DCPs to the patient health record.

After all, we are given individual login IDs. It should not be accessible to unauthorized people, but to those who go there, (sign in) because of their profession. To take a stand on something, that you don't go there out of curiosity. (A7).

If it feels that they are asking questions about a private matter, you can always call. There must be a tactile element involved when the person posing the questions is someone other than this patient of ours. (A2).

Us professionals must be accurate when connecting the patient to the right care path. That we don't accidentally do it incorrectly because there might be a security risk. (A3).

HCPs were aware that use of the digital service was completely voluntary for patients. The participants considered respecting the patient to be important and, as such, counselling via DCPs had to be confidential and individual. The use of evidence‐based information was also important because several professionals were participating in the counselling. The HCPs reported several common practices that were in place to overcome challenging situations.

We have patients who have never gone there. And they do not want to go. Well, then we will take care of things in another way. (A11).

It would create that feeling for the patient, he will be cared for, and his case taken care of. I'm sure that's the goal, but that the human voice would remain there. (A2).

#### 3.2.7 The implementation of digital care pathways requires development competence in developing services

Nursing expertise and technological skills were emphasized when discussing the production of content for DCPs. Skills to support the involvement of users were also necessary.

Expertise for content production was related to the patient group (professional skills), illness‐specific knowledge, and selecting the correct treatment processes. Identifying challenges related to the patient group was an important part of content production. Multidisciplinary expertise, along with utilizing the competence of colleagues and other occupational groups when developing and testing DCPs, were also considered important.

Professional and clinical competence in the topic of the Digital Care Pathway, those particular patient groups and their treatment pathways. (A5).

What can you think the families need and what has been asked in the pre‐visit phone calls. Those are questions which we use to find answers. (A4).

All occupational groups participate according to their own capabilities and their know‐how. (A12).

Producing content for DCPs required a special type of technological knowledge. For example, HCPs need to know what kind of material can be produced for DCPs so that it will be accessible to different patient groups.

(Using) Power Point and Word and making links and hyperlinks. (A4).

Counseling should be accessible in such a way that different customer groups are considered. (A3).

Patients were involved by asking about their interest in the service; furthermore, they were asked to test the DCPs and given the possibility to provide feedback. HCPs needed expertise to utilize this feedback in the development of digital services. The participants felt that an important skill was introducing other HCPs to use DCPs; this involved skills in cooperation, interaction, and motivation.

I taught at the same time as the digital pathway was tested. And then we gave few of these lectures. And then, I have been sitting next to a colleague and have just talked about the DCP in private. And then, of course, I am always available. (A10).

#### 3.2.8 The implementation of digital care pathways requires change competence

Change competence was relevant to the implementation of DCPs. This required a positive attitude towards change, and an awareness of the significance of counselling via DCPs.

HCPs were motivated by the changes brought about by DCPs. Counselling via DCPs required continuous and wide‐ranging learning. A positive attitude towards learning new things made the change easier.

I am a very optimistic character. I believe that developing will continue and then there will be these new patient groups. That is what we are waiting for. (A7).

However, I've already used so many of these, and got used to using different kind of programs and applications. It doesn't have anything to do with the fact that I probably won't learn it or that it's probably very difficult. (A9).

HCPs were aware of the importance of DCPs for health care from both the patients' and professionals' perspectives. Counselling via DCPs was considered useful because it was a new method for contacting and sharing information. As such, this service allowed easy communication between the patient and the professional. The participants also realized the benefits of remote appointments, although most of the respondents had no experience of them.

It motivates us to practice and participate. Because you know, it is increasingly the future. (A10).

There are links which we can guide the parents to explore. So, I can take advantage (of them) in counseling. (A6).

You can participate in some counseling even if you come from somewhere 200 kilometers away. That (the traditional approach) makes no sense when it can be handled in this way. (A12).

## 4 DISCUSSION

The results indicate that HCPs experienced counselling competence in DCPs as a multifaceted entity which requires diverse competences. The performed analysis identified eight areas of competence related to counselling in DCPs, namely, counselling competence related to the use of DCPs, supporting patients' self‐care, information technology competence, competence in creating an interactive counselling relationship via DCPs, information management, ethical competence related to counselling via DCPs, implementation of DCPs requires competence in developing services, and change competence.

Existing digital competence frameworks focus on the development of basic information technology skills, the management of data in electronic patient information systems, digital communication skills, along with awareness of the ethical, legal, privacy and security implications of technology (Nazeha et al., 2020). These competences were also emphasized in the results of this study. Sufficient information technology competence facilitates counselling via DCPs, and HCPs must understand how to manage the information resulting from counselling. Previous studies have confirmed this result. For instance, HCPs with strong IT skills were more likely to utilize digital technology in their work (Nazeha et al., 2020), be positive about online interaction, know how to use patient portals and understand when it was suitable to contact patients (Laukka et al., 2020). It should be acknowledged that HCPs need special technological skills to successfully participate in the development of DCPs. In conclusion, counselling via DCPs requires HCPs to be both professionally and technologically adept.

Ethical competence was another subject that was reflected in both the implementation of counselling via DCP and the development of the service. Ethical challenges related to the digitalization of health care have been identified in previous literature reviews. Nittari et al. (2020) divided these challenges into factors related to the patient's informed consent, data protection and confidentiality, malpractice, and legislation. Jokinen et al. (2021), on the other hand, approached the topic from the patient's point of view. Their research highlighted four themes: beneficence; nonmaleficence; justice; and trust in digital services. According to the results of this study, respect for the patient serves as the foundation of counselling, even though it was carried out in a new, digital environment. This research also identified some new challenges. These were mainly related to ensuring patient safety, data privacy, and patient security during counselling. These ethical issues identified new competencies that are necessary for effective counselling via DCPs, and are touched upon in the following sections.

Firstly, the study produced new information on which counselling competences HCPs need to possess if they are to interact with patients via DCPs. In addition to counselling competence related to self‐care, HCPs must demonstrate counselling competence related to the use of the service. In practice, messages sent by the patient, information related to their symptoms, and questions related to treatment were assessed according to their professional role. Clinical competence and patient involvement skills were essentials for providing patient‐centered counselling via DCPs. Laukka et al., (2020) stated that the role of the HCP will remain unchanged as interactions move online. On the other hand, counselling via DCPs requires a new perspective on assessing patients' competence. The independent use of eHealth services requires patients to have varied skills (Alam et al., 2019; Jokinen et al., 2021) and motivation to familiarize themselves with the service (Jokinen et al., 2021). Patients need certain equipment to access the service (Jokinen et al., 2021), and a functional Internet connection (Alam et al., 2019; Jokinen et al., 2021). If the patient's readiness to use eHealth services is not considered, inequality among vulnerable patient groups may increase within health care (Alam et al., 2019; Jokinen et al., 2021). Furthermore, investments must be made into the training of HCP to ensure patient‐oriented counselling in the use of digital services (Jokinen et al., 2021). Moreover, the way in which health care organizations implement eHealth practices, along with the usability of the service, may strongly impact the use of patient portals. The support targeted at HCPs will work to increase the amount of counselling available through eHealth services (Hörhammer et al., 2021).

Secondly, the participating HCPs emphasized that competence in interactive counselling relationships provided via DCPs and knowledge management skills are the foundation for patient‐centered counselling. DCPs are a new channel for transferring patient‐generated information to the treating unit, so HCPs need novel communication skills in the digital environment. Although communication via DCPs was informal, the lack of nonverbal communication created certain challenges. According to Lee et al., (2020), the tone of responses from HCPs varies from the relevant exchange of information to individual support and relationship building. The challenge is that patients can interpret a HCP's messages in their own way (Laukka et al., 2020; Lee et al., 2020). Therefore, professional skills play a crucial role in communication via DCPs. In addition to assessing the patient's understanding, an HCP must choose the necessary counselling method. It is important to be aware of what kinds of situations counselling via DCPs is suitable for. In this study, the participating HCPs questioned the suitability for emotional support, intimate matters and challenges related to patient identification. Lee et al., (2020) identified situations that were suitable for secure messaging, such as routine medication or sample results issues. On the other hand, acute situations requiring immediate evaluation, emotional support, or answering several questions were not suitable for the digital environment (Lee et al., 2020). Previous studies also found that challenging situations require counselling at the health centre or by phone (Laukka et al., 2020; Lee et al., 2020), and multidisciplinary competences have been highlighted (Laukka et al., 2020). The assessment of the data required professional skills to ensure appropriate counselling and to avoid possible malpractice. The use of patient information improved the quality of counselling via DCPs because this practice made the process more patient‐oriented. According to previous research, it is necessary that HCPs understand the purpose, infrastructure, use and storage of electronic health information (Nazeha et al., 2020). Therefore, awareness of data protection and security challenges is essential. The data protection of eHealth services is influenced by the underlying technology and the HCPs' activities (Nittari et al., 2020), which was also reflected in the results of this study.

Thirdly, the implementation of DCPs requires change competence and skills related to the development of services, both which involve considering the needs of professionals and patient groups. This requires strong professional competence from HCPs. Moreover, HCPs working in digital environments need technological skills related to content production. According to Hulter et al., (2020), the implementation of patient portals in a hospital environment requires not only technological implementation, but also diverse interaction between users. Implementation includes three steps, namely, informing patients and professionals about the portal, incorporating a portal into the practices of professionals, and addressing the needs of patients in the ongoing development of the service. The phases were also shown in the results of this study because HCPs were found to be key to counselling patients in how to use DCPs and introducing other professionals to the service. Patient involvement was considered important, but adapting patient portals to meet patient needs was considered challenging in a hospital environment (Hulter et al., 2020). In this study, the involvement of patients in development was also perceived to be important, but nevertheless limited in practice. Feedback was collected from patients who had tested the DCPs but, for example, workshops were not carried out. It remained unclear whether the reason was a lack of resources, challenges in networking or a lack of know‐how.

### 4.1 Study strengths and limitations

The trustworthiness of the presented research was evaluated based on the criteria of Lincoln and Cuba. The model comprises concepts such as credibility, dependability, confirmability, authenticity, and transferability (Kyngäs et al., 2020; Polit & Beck, 2017). The participants' and researcher's experience with the subject under study strengthened credibility. The researcher based the interview topics on previous research and decided to pilot test the created guide; both of these decisions improved credibility. The use of direct quotes also increased the credibility of the research. To ensure confirmability, the researcher kept a research diary during the research and field notes after each interview. The transferability was improved by using convenience and snowball sampling and reporting the demographic information of the study participants. A detailed description of the analytical process improved the dependability of the data analysis, while the results of the analysis were reviewed together with the last author (blinded for the review).

Even though the sample size was small, the collected data represented the topic well because data saturation was achieved. The study also had some limitations, especially regarding the sample. Most of the participants worked in long‐term care DCPs so there were limited descriptions of the competences necessary for short‐term treatment or therapy DCPs. Furthermore, most of the respondents were registered nurses, which means that the views of other occupational groups remain unclear. The fact that interviews had to be remotely conducted due to the ongoing COVID‐19 pandemic also caused some limitations, as the researchers were more familiar with face‐to‐face interviews and may have missed certain subtle body language. Moreover, it is important to note that the researcher knew some of the participants, but sought to act objectively during the interviews.

## 5 CONCLUSION

This study provides new information about which counselling competences HCPs need to participate in DCPs. HCPs play an essential role in the execution of counselling via DCPs and in the continuous development of this service. In addition to supporting patients' self‐care, HCPs must clearly understand how to use the implemented DCPs. This is a cornerstone of patient‐centered counselling, which is widely regarded as the most appropriate form of patient care. Professional expertise and digital competence are required when HCPs provide counselling via DCPs. Competence in fostering interactive counselling relationships and information management skills are also necessary in patient‐centered counselling. When discussing ethically challenging situations, the participating HCPs emphasized interaction skills, the utilization of evidence‐based information, and multidisciplinary competence. The development of DCPs requires innovation and the consideration of different user groups so the service can provide patients with the maximum benefits.

## 6 RELEVANCE TO CLINICAL PRACTICE

At its best, counselling via DCPs benefits the work of HCPs and the everyday lives of patients. However, to reduce inequality, vulnerable patients must be identified. HCPs must have sufficient competence to ensure effective and safe counselling for different patient groups. The present study provides ample evidence that this topic must be included in the basic education and practical training of HCPs. Furthermore, adequate resources must be allocated to health care in order to ensure adequate competence among HCPs. In addition, HCPs must have sufficient time to provide counselling via DCPs and develop effective, patient‐centered services. Cooperation between different user groups, including patients, is significant, but without competent HCPs it would be impossible to develop and implement counselling via DCPs.

## AUTHOR CONTRIBUTIONS

Made substantial contributions to conception and design, or acquisition of data, or analysis and interpretation of data: JK, AO, PL; Involved in drafting the manuscript or revising it critically for important intellectual content: JK, AO, PL, PK, MR. Agreed to be accountable for all aspects of the work in ensuring that questions related to the accuracy or integrity of any part of the work are appropriately investigated and resolved: JK, AO, PL, PK, MR. Given final approval of the version to be published: JK, AO, PL, PK, MR.

## FUNDING INFORMATION

No funding.

## CONFLICT OF INTEREST STATEMENT

No conflicts of interest.

## CONSENT STATEMENT

The research involved no patients and any invasive intervention on human participants for whom a special ethical consent was required. However, all the study participants had to agree to and return a consent form for participation.

## ETHICS STATEMENT

According to legislation in Finland, Research Ethics Committee approval was not required since the study does not involve minors, direct or indirect physical or physiological harm to the participants, or clinical trials. (Medical Research Act No. 488/1999).

## Supporting information


Data S1.

## Data Availability

All the data have been used in this study.
